# *catena*-Poly[[[dibromidocadmium]-μ_2_-1,1′-(butane-1,4-di­yl)bis­(pyridinium-4-carboxyl­ate)] monohydrate]

**DOI:** 10.1107/S160053681101662X

**Published:** 2011-05-07

**Authors:** Feijun Guo, Yuan Li, Simin Yang, Ruizhan Chen

**Affiliations:** a The Institute of Higher Education, Changchun Normal University, Changchun 130032, People’s Republic of China; b College of Chemistry, Changchun Normal University, Changchun 130032, People’s Republic of China

## Abstract

In the title compound, {[CdBr_2_(C_16_H_16_N_2_O_4_)]·H_2_O}_*n*_, the Cd^II^ ion is six-coordinated by a Br_2_O_4_ donor set, with four O atoms from two bridging 1,1′-(butane-1,4-di­yl)bis­(pyridinium-4-carboxyl­ate) ligands. The ligands link the Cd^II^ ions into a zigzag chain extending along [0
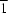
1]. O—H⋯O and O—H⋯Br hydrogen bonds involving the uncoordinated water mol­ecules connect the chains.

## Related literature

For the design and synthesis of coordination polymers, see: Li *et al.* (2005 ▶). For a related structure, see: Ma *et al.* (2000 ▶).
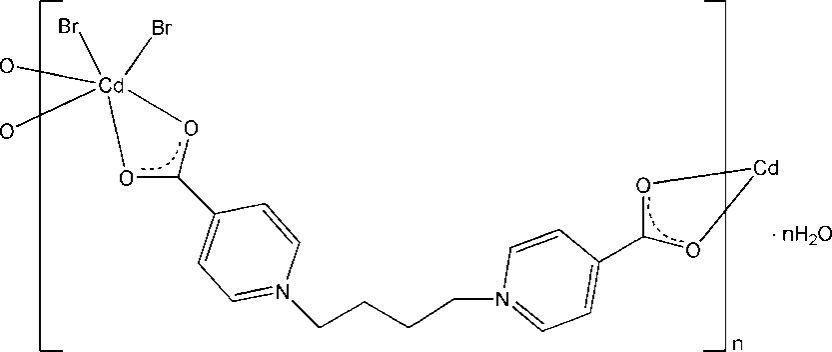


## Experimental

### 

#### Crystal data

[CdBr_2_(C_16_H_16_N_2_O_4_)]·H_2_O
*M* *_r_* = 590.53
Triclinic, 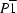

*a* = 7.6529 (5) Å
*b* = 9.3969 (6) Å
*c* = 14.0198 (9) Å
α = 74.410 (1)°
β = 87.060 (2)°
γ = 71.581 (1)°
*V* = 920.75 (10) Å^3^
*Z* = 2
Mo *K*α radiation
μ = 5.56 mm^−1^
*T* = 296 K
0.20 × 0.17 × 0.16 mm

#### Data collection

Bruker APEX CCD diffractometer
Absorption correction: multi-scan (*SADABS*; Sheldrick, 1996 ▶) *T*_min_ = 0.34, *T*_max_ = 0.41
5114 measured reflections
3600 independent reflections
3238 reflections with *I* > 2σ(*I*)
*R*_int_ = 0.013

#### Refinement

*R*[*F* ^2^ > 2σ(*F* ^2^)] = 0.042
*wR*(*F* ^2^) = 0.118
*S* = 1.08
3600 reflections
241 parameters
4 restraints
H atoms treated by a mixture of independent and constrained refinement
Δρ_max_ = 1.45 e Å^−3^
Δρ_min_ = −2.11 e Å^−3^

### 

Data collection: *SMART* (Bruker, 2007 ▶); cell refinement: *SAINT* (Bruker, 2007 ▶); data reduction: *SAINT*; program(s) used to solve structure: *SHELXS97* (Sheldrick, 2008 ▶); program(s) used to refine structure: *SHELXL97* (Sheldrick, 2008 ▶); molecular graphics: *SHELXTL* (Sheldrick, 2008 ▶) and *DIAMOND* (Brandenburg, 1999 ▶); software used to prepare material for publication: *SHELXTL*.

## Supplementary Material

Crystal structure: contains datablocks global, I. DOI: 10.1107/S160053681101662X/hy2426sup1.cif

Structure factors: contains datablocks I. DOI: 10.1107/S160053681101662X/hy2426Isup2.hkl

Additional supplementary materials:  crystallographic information; 3D view; checkCIF report

## Figures and Tables

**Table 1:** Selected bond lengths (Å)

Cd1—O1	2.476 (4)
Cd1—O2	2.345 (4)
Cd1—O3^i^	2.409 (4)
Cd1—O4^i^	2.435 (4)
Cd1—Br1	2.5728 (8)
Cd1—Br2	2.6162 (8)

Symmetry code: (i) 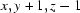.

**Table 2:** Hydrogen-bond geometry (Å, °)

*D*—H⋯*A*	*D*—H	H⋯*A*	*D*⋯*A*	*D*—H⋯*A*
O1*W*—H1*A*⋯O2^ii^	0.84 (7)	2.10 (7)	2.934 (6)	174 (5)
O1*W*—H1*B*⋯Br2^iii^	0.83 (5)	2.65 (6)	3.467 (4)	169 (6)

Symmetry codes: (ii) 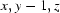; (iii) 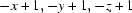.

**Fig. 1.**
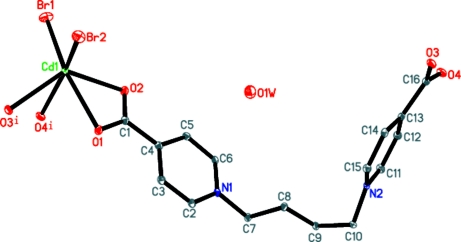
The asymmetric unit of the title compound. Displacement ellipsoids are drawn at the 30% probability level. H atoms have been omitted for clarity. [Symmetry code: (i) x, 1+y, -1+z.]

**Fig. 2.**
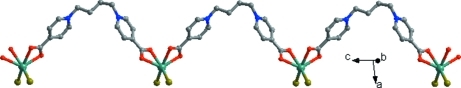
View of the zigzag chain.

## supplementary crystallographic information

### Comment

The design and synthesis of coordination polymers are of great interest for their intriguing architectures and potential applications (Li *et al.*, 2005). In this paper, the structure of the title compound is described.

As shown in Fig. 1, the Cd^II^ ion is six-coordinated by two Br^-^ anions and four O atoms from two butane-1,4-diylbis(pyridinium-1-yl-4-carboxylate (*L*) ligands (Table 1). The two carboxylate groups of the *L* ligand display a bidentate chelating mode. The bond distances and angles are normal (Ma *et al.*, 2000). As illustrated in Fig. 2, each *L* ligand bridges two Cd^II^ ions, resulting in a one-dimensional zigzag chain, with a Cd···Cd separation of 14.630 (1) Å. O—H···O and O—H···Br hydrogen bonds (Table 2) involving the uncoordinated water molecules connect the chains.

### Experimental

The ligand *L* was synthesized according to literature (Li *et al.*, 2005). A mixture of Cd(NO_3_)_2_.4H_2_O (0.015 g), *L* (0.023 g), NaOH (0.004 g) and water (10 ml) was heated at 80°C for 25 min. After the mixture had been cooled to room temperature, colorless crystals of the title compound were obtained (yield: 43%).

### Refinement

H atoms on C atoms were positioned geometrically and refined as riding atoms, with C—H = 0.93 (CH) and 0.97 (CH_2_) Å and with *U*_iso_(H)= 1.2*U*_eq_(C). Water H atoms were located in a difference Fourier map and refined with a restraint of O—H = 0.84 (1) Å and with *U*_iso_(H) = 1.5*U*_eq_(O). The highest residual electron density was found at 0.33 Å from Cd1 atom and the deepest hole at 0.37 Å from Br2 atom.

### Crystal data

**Table:**

[CdBr_2_(C_16_H_16_N_2_O_4_)]·H_2_O	*Z* = 2
*M**_r_* = 590.53	*F*(000) = 572
Triclinic, *P*1	*D*_x_ = 2.130 Mg m^−^^3^
Hall symbol: -P 1	Mo *K*α radiation, λ = 0.71073 Å
*a* = 7.6529 (5) Å	Cell parameters from 3238 reflections
*b* = 9.3969 (6) Å	θ = 1.5–26.2°
*c* = 14.0198 (9) Å	µ = 5.56 mm^−^^1^
α = 74.410 (1)°	*T* = 296 K
β = 87.060 (2)°	Block, colorless
γ = 71.581 (1)°	0.20 × 0.17 × 0.16 mm
*V* = 920.75 (10) Å^3^	

### Data collection

**Table:**

Bruker APEX CCD diffractometer	3600 independent reflections
Radiation source: fine-focus sealed tube	3238 reflections with *I* > 2σ(*I*)
graphite	*R*_int_ = 0.013
φ and ω scans	θ_max_ = 26.2°, θ_min_ = 1.5°
Absorption correction: multi-scan (*SADABS*; Sheldrick, 1996)	*h* = −9→9
*T*_min_ = 0.34, *T*_max_ = 0.41	*k* = −7→11
5114 measured reflections	*l* = −17→17

### Refinement

**Table:**

Refinement on *F*^2^	Primary atom site location: patterson
Least-squares matrix: full	Secondary atom site location: difference Fourier map
*R*[*F*^2^ > 2σ(*F*^2^)] = 0.042	Hydrogen site location: inferred from neighbouring sites
*wR*(*F*^2^) = 0.118	H atoms treated by a mixture of independent and constrained refinement
*S* = 1.08	*w* = 1/[σ^2^(*F*_o_^2^) + (0.0524*P*)^2^ + 9.2463*P*] where *P* = (*F*_o_^2^ + 2*F*_c_^2^)/3
3600 reflections	(Δ/σ)_max_ = 0.001
241 parameters	Δρ_max_ = 1.45 e Å^−^^3^
4 restraints	Δρ_min_ = −2.11 e Å^−^^3^

### Fractional atomic coordinates and isotropic or equivalent isotropic displacement parameters (Å^2^)

**Table:**

	*x*	*y*	*z*	*U*_iso_*/*U*_eq_	
Cd1	0.47375 (5)	1.16457 (5)	0.24196 (3)	0.01263 (13)	
C1	0.7499 (8)	0.9542 (7)	0.3709 (4)	0.0134 (11)	
C2	1.1656 (8)	0.6169 (7)	0.4914 (4)	0.0131 (11)	
H4	1.2521	0.5317	0.4764	0.016*	
C3	1.0287 (8)	0.7160 (7)	0.4228 (4)	0.0149 (11)	
H3	1.0244	0.6995	0.3606	0.018*	
C4	0.8966 (8)	0.8412 (7)	0.4467 (4)	0.0130 (11)	
C5	0.9054 (8)	0.8616 (7)	0.5403 (4)	0.0142 (11)	
H6	0.8164	0.9424	0.5585	0.017*	
C6	1.0467 (8)	0.7616 (6)	0.6065 (4)	0.0129 (11)	
H5	1.0537	0.7760	0.6691	0.015*	
C7	1.3272 (8)	0.5417 (7)	0.6535 (4)	0.0136 (11)	
H7A	1.3690	0.6045	0.6861	0.016*	
H7B	1.4298	0.4894	0.6187	0.016*	
C8	1.2649 (8)	0.4214 (7)	0.7305 (4)	0.0144 (11)	
H8A	1.2521	0.3430	0.7008	0.017*	
H8B	1.1457	0.4711	0.7539	0.017*	
C9	1.4043 (8)	0.3444 (7)	0.8174 (4)	0.0135 (11)	
H9A	1.5276	0.3192	0.7923	0.016*	
H9B	1.3945	0.4173	0.8565	0.016*	
C10	1.3755 (8)	0.1972 (7)	0.8834 (4)	0.0135 (11)	
H10A	1.3859	0.1243	0.8442	0.016*	
H10B	1.4723	0.1495	0.9349	0.016*	
C11	1.1690 (8)	0.2881 (7)	1.0082 (4)	0.0157 (12)	
H11	1.2593	0.3246	1.0254	0.019*	
C12	1.0140 (8)	0.2988 (7)	1.0617 (4)	0.0154 (11)	
H12	0.9988	0.3416	1.1153	0.019*	
C13	0.8779 (8)	0.2448 (6)	1.0353 (4)	0.0127 (11)	
C14	0.9010 (8)	0.1903 (7)	0.9516 (4)	0.0138 (11)	
H14	0.8086	0.1604	0.9299	0.017*	
C15	1.0608 (8)	0.1803 (7)	0.9003 (4)	0.0144 (11)	
H15	1.0771	0.1422	0.8446	0.017*	
C16	0.7200 (8)	0.2339 (7)	1.1034 (4)	0.0146 (11)	
N1	1.1736 (7)	0.6442 (6)	0.5809 (3)	0.0123 (9)	
N2	1.1936 (6)	0.2255 (5)	0.9307 (3)	0.0103 (9)	
O1	0.7496 (6)	0.9313 (5)	0.2877 (3)	0.0158 (8)	
O2	0.6345 (6)	1.0670 (5)	0.3958 (3)	0.0169 (9)	
O3	0.6036 (6)	0.1791 (5)	1.0807 (3)	0.0171 (9)	
O4	0.7215 (6)	0.2727 (5)	1.1822 (3)	0.0181 (9)	
Br1	0.24542 (9)	1.43346 (8)	0.24021 (6)	0.0306 (2)	
Br2	0.26563 (10)	0.98804 (8)	0.25061 (5)	0.02913 (19)	
O1W	0.4919 (7)	0.2586 (5)	0.5341 (3)	0.0234 (10)	
H1A	0.529 (11)	0.199 (8)	0.497 (5)	0.035*	
H1B	0.539 (11)	0.207 (9)	0.590 (3)	0.035*	

### Atomic displacement parameters (Å^2^)

**Table:**

	*U*^11^	*U*^22^	*U*^33^	*U*^12^	*U*^13^	*U*^23^
Cd1	0.0110 (2)	0.0143 (2)	0.0108 (2)	−0.00276 (16)	0.00083 (14)	−0.00187 (15)
C1	0.015 (3)	0.014 (3)	0.013 (3)	−0.008 (2)	0.001 (2)	−0.002 (2)
C2	0.015 (3)	0.014 (3)	0.012 (3)	−0.006 (2)	0.001 (2)	−0.004 (2)
C3	0.019 (3)	0.016 (3)	0.011 (3)	−0.007 (2)	0.001 (2)	−0.005 (2)
C4	0.013 (3)	0.014 (3)	0.014 (3)	−0.007 (2)	0.001 (2)	−0.003 (2)
C5	0.015 (3)	0.014 (3)	0.014 (3)	−0.004 (2)	0.004 (2)	−0.005 (2)
C6	0.017 (3)	0.011 (3)	0.010 (3)	−0.004 (2)	0.003 (2)	−0.003 (2)
C7	0.012 (3)	0.017 (3)	0.011 (3)	−0.005 (2)	−0.002 (2)	0.000 (2)
C8	0.011 (3)	0.014 (3)	0.016 (3)	−0.006 (2)	−0.001 (2)	0.001 (2)
C9	0.013 (3)	0.015 (3)	0.011 (3)	−0.005 (2)	0.001 (2)	0.000 (2)
C10	0.011 (3)	0.015 (3)	0.012 (3)	−0.002 (2)	0.003 (2)	−0.003 (2)
C11	0.016 (3)	0.020 (3)	0.013 (3)	−0.009 (2)	0.000 (2)	−0.004 (2)
C12	0.017 (3)	0.017 (3)	0.013 (3)	−0.004 (2)	0.001 (2)	−0.005 (2)
C13	0.014 (3)	0.009 (3)	0.012 (3)	−0.002 (2)	0.000 (2)	−0.001 (2)
C14	0.016 (3)	0.013 (3)	0.011 (3)	−0.005 (2)	−0.003 (2)	−0.001 (2)
C15	0.015 (3)	0.014 (3)	0.014 (3)	−0.005 (2)	0.000 (2)	−0.003 (2)
C16	0.015 (3)	0.012 (3)	0.014 (3)	−0.002 (2)	0.001 (2)	−0.002 (2)
N1	0.015 (2)	0.012 (2)	0.010 (2)	−0.0066 (19)	0.0010 (18)	−0.0004 (18)
N2	0.012 (2)	0.010 (2)	0.008 (2)	−0.0035 (18)	0.0005 (17)	0.0000 (17)
O1	0.017 (2)	0.017 (2)	0.012 (2)	−0.0024 (17)	−0.0020 (16)	−0.0040 (16)
O2	0.020 (2)	0.017 (2)	0.012 (2)	−0.0018 (17)	−0.0011 (16)	−0.0040 (16)
O3	0.014 (2)	0.026 (2)	0.012 (2)	−0.0086 (18)	0.0017 (15)	−0.0031 (17)
O4	0.017 (2)	0.025 (2)	0.015 (2)	−0.0080 (18)	0.0052 (16)	−0.0081 (18)
Br1	0.0196 (3)	0.0228 (4)	0.0477 (5)	−0.0041 (3)	0.0005 (3)	−0.0095 (3)
Br2	0.0287 (4)	0.0294 (4)	0.0303 (4)	−0.0093 (3)	0.0054 (3)	−0.0102 (3)
O1W	0.030 (3)	0.018 (2)	0.020 (2)	−0.005 (2)	0.0032 (19)	−0.0052 (18)

### Geometric parameters (Å, °)

**Table:**

Cd1—O1	2.476 (4)	C8—H8A	0.9700
Cd1—O2	2.345 (4)	C8—H8B	0.9700
Cd1—O3^i^	2.409 (4)	C9—C10	1.518 (8)
Cd1—O4^i^	2.435 (4)	C9—H9A	0.9700
Cd1—Br1	2.5728 (8)	C9—H9B	0.9700
Cd1—Br2	2.6162 (8)	C10—N2	1.487 (7)
C1—O1	1.241 (7)	C10—H10A	0.9700
C1—O2	1.266 (7)	C10—H10B	0.9700
C1—C4	1.510 (8)	C11—N2	1.347 (7)
C2—N1	1.354 (7)	C11—C12	1.361 (8)
C2—C3	1.377 (8)	C11—H11	0.9300
C2—H4	0.9300	C12—C13	1.398 (8)
C3—C4	1.394 (8)	C12—H12	0.9300
C3—H3	0.9300	C13—C14	1.385 (8)
C4—C5	1.383 (8)	C13—C16	1.513 (8)
C5—C6	1.380 (8)	C14—C15	1.376 (8)
C5—H6	0.9300	C14—H14	0.9300
C6—N1	1.338 (7)	C15—N2	1.345 (7)
C6—H5	0.9300	C15—H15	0.9300
C7—N1	1.491 (7)	C16—O3	1.252 (7)
C7—C8	1.517 (8)	C16—O4	1.254 (7)
C7—H7A	0.9700	O1W—H1A	0.84 (7)
C7—H7B	0.9700	O1W—H1B	0.83 (5)
C8—C9	1.522 (8)		
			
O2—Cd1—O3^i^	127.10 (14)	C8—C7—H7B	109.5
O2—Cd1—O4^i^	86.07 (15)	H7A—C7—H7B	108.1
O3^i^—Cd1—O4^i^	54.49 (14)	C7—C8—C9	110.3 (5)
O2—Cd1—O1	54.51 (14)	C7—C8—H8A	109.6
O3^i^—Cd1—O1	81.38 (14)	C9—C8—H8A	109.6
O4^i^—Cd1—O1	78.10 (14)	C7—C8—H8B	109.6
O2—Cd1—Br1	106.91 (10)	C9—C8—H8B	109.6
O3^i^—Cd1—Br1	108.36 (11)	H8A—C8—H8B	108.1
O4^i^—Cd1—Br1	92.51 (10)	C10—C9—C8	112.4 (5)
O1—Cd1—Br1	159.25 (10)	C10—C9—H9A	109.1
O2—Cd1—Br2	104.34 (11)	C8—C9—H9A	109.1
O3^i^—Cd1—Br2	103.49 (10)	C10—C9—H9B	109.1
O4^i^—Cd1—Br2	156.16 (10)	C8—C9—H9B	109.1
O1—Cd1—Br2	90.43 (10)	H9A—C9—H9B	107.8
Br1—Cd1—Br2	104.48 (3)	N2—C10—C9	113.1 (5)
O2—Cd1—C16^i^	106.48 (16)	N2—C10—H10A	108.9
O3^i^—Cd1—C16^i^	27.25 (16)	C9—C10—H10A	108.9
O4^i^—Cd1—C16^i^	27.32 (16)	N2—C10—H10B	108.9
O1—Cd1—C16^i^	76.82 (15)	C9—C10—H10B	108.9
Br1—Cd1—C16^i^	103.21 (12)	H10A—C10—H10B	107.8
Br2—Cd1—C16^i^	129.79 (12)	N2—C11—C12	121.1 (5)
O2—Cd1—C1	27.52 (16)	N2—C11—H11	119.5
O3^i^—Cd1—C1	104.69 (16)	C12—C11—H11	119.5
O4^i^—Cd1—C1	81.35 (16)	C11—C12—C13	119.4 (5)
O1—Cd1—C1	26.99 (15)	C11—C12—H12	120.3
Br1—Cd1—C1	133.92 (12)	C13—C12—H12	120.3
Br2—Cd1—C1	97.91 (12)	C14—C13—C12	118.4 (5)
C16^i^—Cd1—C1	91.88 (17)	C14—C13—C16	122.0 (5)
O1—C1—O2	123.8 (5)	C12—C13—C16	119.3 (5)
O1—C1—C4	118.2 (5)	C15—C14—C13	120.0 (5)
O2—C1—C4	118.0 (5)	C15—C14—H14	120.0
O1—C1—Cd1	64.9 (3)	C13—C14—H14	120.0
O2—C1—Cd1	58.9 (3)	N2—C15—C14	120.1 (5)
C4—C1—Cd1	176.8 (4)	N2—C15—H15	120.0
N1—C2—C3	119.7 (5)	C14—C15—H15	120.0
N1—C2—H4	120.2	O3—C16—O4	124.4 (6)
C3—C2—H4	120.2	O3—C16—C13	118.5 (5)
C2—C3—C4	119.9 (5)	O4—C16—C13	116.9 (5)
C2—C3—H3	120.1	O3—C16—Cd1^ii^	61.8 (3)
C4—C3—H3	120.1	O4—C16—Cd1^ii^	63.0 (3)
C5—C4—C3	118.7 (5)	C13—C16—Cd1^ii^	170.2 (4)
C5—C4—C1	120.9 (5)	C6—N1—C2	121.7 (5)
C3—C4—C1	120.4 (5)	C6—N1—C7	118.6 (5)
C6—C5—C4	119.7 (5)	C2—N1—C7	119.8 (5)
C6—C5—H6	120.1	C15—N2—C11	120.8 (5)
C4—C5—H6	120.1	C15—N2—C10	120.3 (5)
N1—C6—C5	120.3 (5)	C11—N2—C10	118.7 (5)
N1—C6—H5	119.9	C1—O1—Cd1	88.1 (3)
C5—C6—H5	119.9	C1—O2—Cd1	93.6 (3)
N1—C7—C8	110.8 (4)	C16—O3—Cd1^ii^	90.9 (3)
N1—C7—H7A	109.5	C16—O4—Cd1^ii^	89.7 (4)
C8—C7—H7A	109.5	H1A—O1W—H1B	105 (8)
N1—C7—H7B	109.5		

Symmetry codes: (i) *x*, *y*+1, *z*−1; (ii) *x*, *y*−1, *z*+1.

### Hydrogen-bond geometry (Å, °)

**Table:**

*D*—H···*A*	*D*—H	H···*A*	*D*···*A*	*D*—H···*A*
O1W—H1A···O2^iii^	0.84 (7)	2.10 (7)	2.934 (6)	174 (5)
O1W—H1B···Br2^iv^	0.83 (5)	2.65 (6)	3.467 (4)	169 (6)

Symmetry codes: (iii) *x*, *y*−1, *z*; (iv) −*x*+1, −*y*+1, −*z*+1.

## References

[bb1] Brandenburg, K. (1999). *DIAMOND.* Crystal Impact GbR, Bonn, Germany.

[bb2] Bruker (2007). *SMART* and *SAINT* Bruker AXS Inc., Madison, Wisconsin, USA.

[bb3] Li, S.-L., Ji, W.-Z., Hou, J.-F. & Tian, D.-K. (2005). *Chin. J. Inorg. Chem.* **1**, 30–34.

[bb4] Ma, J.-F., Liu, J.-F., Xing, Y., Jia, H.-Q. & Lin, Y.-H. (2000). *J. Chem. Soc. Dalton Trans.* pp. 2403–2407.

[bb5] Sheldrick, G. M. (1996). *SADABS* University of Göttingen, Germany.

[bb6] Sheldrick, G. M. (2008). *Acta Cryst.* A**64**, 112–122. 10.1107/S0108767307043930 18156677

